# Different green synthesis methods of Co_3_O_4_ NPs using aloe vera leaves: enhance H_2_ and O_2_ production from NaBH_4_ hydrolysis and H_2_O_2_ decomposition

**DOI:** 10.1038/s41598-026-62856-x

**Published:** 2026-07-24

**Authors:** Simon W. Samouel, Tarek T. Ali, Bahaa M. Abu-Zied, Hatem A. Mahmoud

**Affiliations:** 1 Chemistry Department, Sohag University, Sohag, 82524 Egypt; 2 Chemistry Department, Assiut University, Assiut, 71516 Egypt

**Keywords:** Green synthesis, Co_3_O_4_ NPs, Hydrothermal, Microwave, Co-precipitation, Aloe vera leaves, Dual function, NaBH_4_ hydrolysis, H_2_O_2_ decomposition, Chemistry, Materials science, Nanoscience and technology

## Abstract

Green synthesis of cobalt oxide nanoparticles (Co_3_O_4_ NPs) was achieved using aloe vera leaves via hydrothermal, microwave irradiation, and co-precipitation methods. XRD analysis confirmed the high purity and crystallinity of the catalysts, as no other phases or impurities were observed in the crystal lattice. Catalysts’ morphology was visualized by FESEM images that showed flower-like morphology for AHT and capsule-like shapes for AMW and AWB. The chemical environment was identified by XPS analysis, which also confirmed the synthesis of Co_3_O_4_ NPs by the presence of Co^2+^ and Co^3+^ ions on the catalysts’ surface. All catalysts exhibited dual functionality: catalyzing NaBH_4_ hydrolysis and H_2_O_2_ decomposition. During NaBH_4_ hydrolysis, AHT demonstrated superior catalytic performance with a hydrogen generation rate (HGR) of 4267 ml.min^− 1^.g^− 1^ at 45 °C. Further study on the efficient catalyst (AHT) was performed to evaluate the effect of catalyst weight, NaBH_4_ concentration, alkalinity, and recyclability on catalytic activity. In H_2_O_2_ decomposition, AWB displayed higher activity with a lower activation energy (E_a_) of 39.66 kJ/mol.

**Supplementary Information:**

The online version contains supplementary material available at 10.1038/s41598-026-62856-x.

## Introduction

Cobalt oxides have a diversity of chemical structures with different oxidation states as Co^2+^ in CoO, Co^3+^ in Co_2_O_3_, and a mixture of Co^2+^ and Co^3+^ in Co_3_O_4_. Cobaltosic oxide nanoparticles (Co_3_O_4_ NPs) are particularly significant in numerous applications compared to other cobalt oxides such as CoO and Co_2_O_3_. Two oxidation states are located in different positions of the spinel structure of Co_3_O_4,_ as Co^3+^ ions at octahedral sites and Co^2+^ ions at tetrahedral sites^1^. The green synthesis of Co_3_O_4_ NPs has been documented through various biological sources, including plants, microorganisms, and other biomolecules^2^. In this context, diverse methodologies such as hydrothermal^3^, microwave irradiation^4^, precipitation^5^, and combustion^6^ are employed for the synthesis of Co_3_O_4_ NPs. The synthesized Co_3_O_4_ NPs derived from green sources demonstrate higher efficiency toward various applications, including antibacterial activity^7^, photocatalytic activity^8,9^, and electrochemical applications. Also, they are used in catalysis applications such as the hydrolysis of NaBH_4_^2,6^ and the decomposition of H_2_O_2_^10^.

In the past years, the world’s energy production relied on fossil fuels, whose resources are reducing, and their extraction and processing contribute to environmental devastation. In the meantime, hydrogen (H_2_) has emerged as a renewable and sustainable energy source due to its high energy density and zero greenhouse gas emissions^11–13^. Sodium borohydride (NaBH_4_) has received substantial attention as a crucial hydrogen storage material for hydrogen generation involving high hydrogen storage capacity (volumetrically: 0.115 kg.H_2_.L^− 1^ and gravimetrically: 10.8 wt% of H_2_)^14–16^, efficient thermal, ideal hydrolysis controllability, chemical stability, and eco-friendliness of the reaction products^17^. Since the 1950 s, Schlesinger et al. reported the first literature on the hydrolysis of NaBH_4_ to generate hydrogen under mild conditions^18^. Meanwhile, self-hydrolysis of NaBH_4_ yields lower hydrogen production at ambient temperature. Therefore, researchers have developed various catalysts to accelerate the hydrolysis reaction^19^. Hydrolysis of NaBH_4_ is a spontaneous exothermic reaction, which can be expressed by the following equation:

1 $$\:{\mathrm{N}\mathrm{a}\mathrm{B}\mathrm{H}}_{4}+{2\mathrm{H}}_{2}\mathrm{O}\to\:\:{\mathrm{N}\mathrm{a}\mathrm{B}\mathrm{O}}_{2}+{4\mathrm{H}}_{2}+\varDelta\:\mathrm{H}$$

Hence, the byproduct from the reaction, such as NaBO_2,_ is non-toxic and can be potentially recycled for the synthesis of NaBH_4_^20^. Noble metals such as platinum^21^, palladium^22^, ruthenium^23^, and silver^24^ have superior catalytic performance and rapid hydrogen generation in this reaction. These metals have less utilization in the NaBH_4_ hydrolysis reaction because of their rarity and high cost. On the contrary, nonprecious metals exhibit lower catalytic activity than noble metals, but they are also low-cost and affordable. The transition metals utilized in this hydrolysis process involve cobalt^1,4^, nickel^25^, and iron^26^. Among the catalysts utilized in the hydrolysis of NaBH_4_, Co_3_O_4_ NPs attract potential attention due to their cost-affordable and high activity performance. Krishnan et al.^27^ reported that synthesized Co_3_O_4_ NPs could have higher catalytic activity by thermal decomposition than some noble metal catalysts, involving Pt/C and Ru/C.

In recent times, hydrogen peroxide (H_2_O_2_) has been regarded as an essential chemical product in industrial chemical processes. It is a distinctive substance due to its molecular structure, comprising oxygen atoms in the oxidation state of −1, unlike many compounds where oxygen exists in oxidation states of 0 or −2. This illustrates that H_2_O_2_ can function as both an oxidizing and a reducing agent, depending on the pH of its solution. Moreover, H_2_O_2_ is a versatile chemical and sustainable energy carrier as a fuel; the only products are oxygen, water, and heat with zero carbon emissions^28,29^. It serves as an eco-friendly oxidant widely used in households, agriculture, medicine, and various industries, including textile bleaching, chemical synthesis, environmental control, and effluent treatment (acting as a substitute for chlorine in water and sewage treatment), as well as sterilization and cleansing^30^. The key active component of H_2_O_2_ is nascent oxygen, which underscores its applications in the aforementioned uses. Active oxygen can be generated through the controlled decomposition of H_2_O_2_, with water as a byproduct. The catalytic activity of H_2_O_2_ can be adjusted or enhanced using co-regents or activator catalysts. The following exothermic reaction is illustrated using a catalyst or an enzyme in the decomposition:

2 $$\:{H}_{2}{O}_{2}^{\underrightarrow{Catalyst/Enzyme}}\:{H}_{2}O+{1/2}\:{O}_{2}\uparrow\:$$

Some factors that affect the rate of reaction are the concentration of the solution, the type of catalyst, temperature, pressure, the surface area of the catalyst, exposure to direct sunlight, and the presence of inhibitors^31^. The reaction of decomposition of H_2_O_2_ is very slow at moderate temperatures without the presence of a catalyst^32^. Several transition metal ions and metal complexes have been used to catalyze this reaction^33,34^. Diverse types of catalysts are utilized in this reaction, such as heterogeneous (e.g., gold, silver, and iron), homogeneous (iron ions or iodide), and enzymes (catalase)^30^. Interestingly, Co_3_O_4_ NPs exhibited superior catalytic performance in the H_2_O_2_ decomposition reaction based on previous literature studies^10,35^.

This study employed various methods for the green synthesis of Co_3_O_4_ NPs to address environmental concerns, including the enhancement of a sustainable energy source (H_2_) and the removal of H_2_O_2_ prior to environmental discharge. Consequently, the catalysts synthesized are used in a dual function for both NaBH_4_ hydrolysis and H_2_O_2_ decomposition reactions. The prepared catalysts were characterized by utilizing several techniques, such as X-ray diffraction (XRD), Fourier-transform infrared (FT-IR), Field electron scanning electron microscopy (FESEM), N_2_- adsorption isotherm, and X-ray photoelectron spectroscopy (XPS). Subsequently, all catalysts were evaluated in both reactions to obtain HGR and OER values.

## Experimental

### Chemicals and materials

#### Plant materials

Aloe vera plants were collected from my home in Sohag Governorate, Egypt, and utilized as a green precursor for synthesizing Co_3_O_4_ nanoparticles for all catalysts.

### Chemical materials

The chemicals were used as received without further purification during the catalyst preparation and activity measurements as follows: Cobalt nitrate (II) hexahydrate (Co(NO_3_)_2_⋅6H_2_O, Alpha Chemika, Assay Min. 99%) was utilized as a metal salt of cobalt in the green synthesis of Co_3_O_4_ NPs catalyst. Sodium borohydride (NaBH_4_, SDFCL, Assay Min. 98%) was utilized in the hydrogen generation system of the catalytic hydrolysis of NaBH_4_. Sodium hydroxide (NaOH, PIOCHEM, Assay 99%) was used as a basic medium in the catalytic hydrolysis of NaBH_4_. Hydrogen peroxide (H_2_O_2_, Sigma Aldrich, conc. 30%) was used in the decomposition reaction. Distilled water was utilized for all the catalyst synthesis and purification.

### Synthesis procedures

#### Preparation of aloe vera extract

Approximately 100 g of fresh plant was washed with tap water and then distilled water to remove any dust and contaminants. Then, a Moulinex chopper was utilized to finely cut the washed parsley leaves for a few minutes. The choppered plant was diluted to 400 ml of distilled water in a round flask and refluxed at 90 °C for 1 h. The extract was permitted to cool and subsequently filtered using Whatman filter paper.

### Synthesis of Co_3_O_4_ NPs by various approaches

Approximately 300 mL of plant extract (yellow color) was taken in a beaker, and 50 ml of Co(NO_3_)_2_.6H_2_O (containing 15 g) was added. Subsequently, the mixture was stirred, and the pH was adjusted to 9 by using an NH_4_OH solution, the color of which was changed to green.

- Hydrothermal approaches: The mixture was divided into two halves, each one being transferred into a 300 ml Teflon-lined stainless-steel autoclave, then heated in an oven at 150 °C for 48 h.
- Microwave approach: The obtained precipitation was exposed to microwave irradiation in SHARP Microwave at 600 W for 8 min.
- Co-precipitation approach: The beaker containing precipitate was transferred into a JSR water bath that was adjusted to 95 °C for 12 h.

**Fig. 1:**
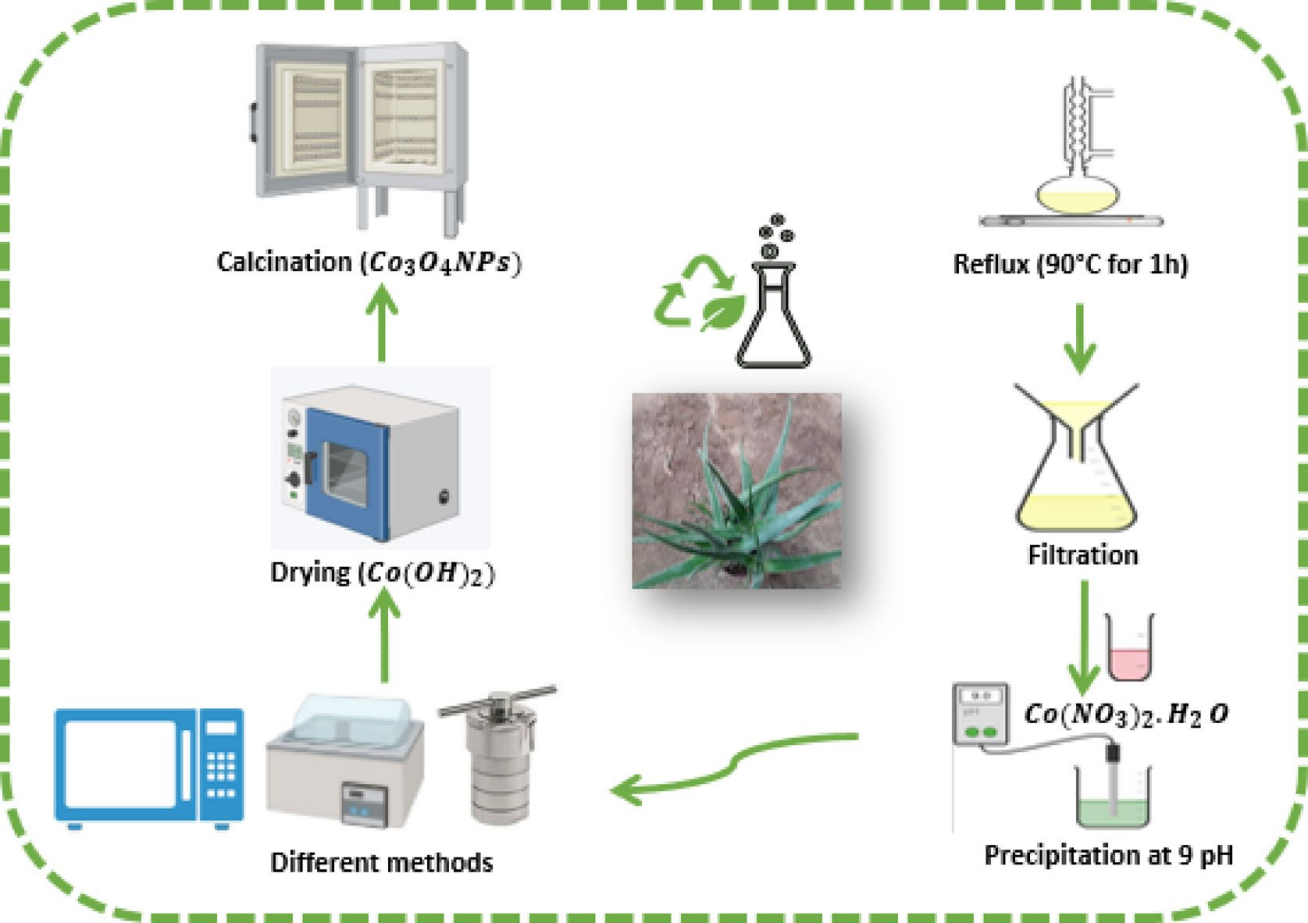
Schematic of experimental procedures for green-synthesized Co_3_O_4_ NPs by using aloe vera extract via various methods (hydrothermal, microwave irradiation, and co-precipitation).

Various preparation methods were employed, whereby the precipitates were washed multiple times with distilled water and ethanol to remove organic residues. Subsequently, the yields were dried overnight in a Heraeus oven set at 80 °C. All prepared samples were subsequently calcined in a muffle furnace at 400 °C for 12 h with a heating rate of 1 °C min^− 1^ under air flow, resulting in black powders that confirmed the successful synthesis of Co_3_O_4_ NPs. Several steps involved during the synthesis of Co_3_O_4_ NPs utilizing aloe vera extract by various methods are schematically presented in Fig. 1.

### Material characterization

At the lab scale, the XRD patterns of the synthesized Co_3_O_4_ NPs were analyzed at Sohag University utilizing a Bruker diffractometer (model D8). The operating parameters included monochromatic Cu-Kα radiation (λ = 1.5418 Å), a scanning rate of 0.05 min^− 1^, and a 2θ range from 15° to 80°. Whereas, Synchrotron XRD measurements were conducted at the MS beamline (ID09) of SESAME synchrotron in Jordan, employing a wavelength of 0.8272 Å (15 keV) with a 2θ range from 2° to 80° and a Pilatus 300 K detector. The Rietveld refinement data were obtained using the FullProf program (FullProf Suite version for windows 2021), and the chemical structure was visualized with Visualization for Electronic and Structural Analysis (VESTA) software (version 3.90.5a for windows 2025). A BRUKER spectrophotometer model ALPHA was used to obtain FT-IR spectra over the 4000–400 cm^− 1^ range. Morphology estimation was visualized with a FESEM (Zeiss Sigma 500 VP) at magnifications from 5 to 80,000 KX, with an accelerating voltage (EHT) of 5.00 kV. The catalysts were coated with gold before measurement to ensure high purity of the scanned NPs. To identify the textural properties and nitrogen adsorption-desorption isotherm of the prepared catalysts, a Quantachrome (model NOVA 3200) gas sorption apparatus was used at −196 °C. Surface assessment using XPS analysis has been performed on a ThermoFisher XPS Spectrometer (model K-alpha). The conditions utilized were monochromatic (X-ray Al K-alpha radiation), spot size 400 μm, binding energy (BE) range between 0 and 1347.8 eV, and pressure of 5 × 10^− 10^ mbar.

### Catalytic system assessment

The catalytic activity experiments were examined through a gasometric apparatus that was similar to a closed-glass apparatus as reported by Deren´ et al.^36^. The apparatus is composed of a 100 ml reaction flask connected to a manometer and two burettes (50 and 100 ml) filled with distilled water. The reaction flask was placed inside a 1 l beaker on a hotplate with a magnetic stirrer, whose temperature is adjusted using a contact thermometer. The volume of hydrogen liberated during the hydrolysis of NaBH_4_ was measured by the water displacement from burettes. The activity run includes 10 mg of the synthesized catalyst and 20 ml of 0.75 wt% NaBH_4_, which was added to the vessel and connected to the glass apparatus. The volume of hydrogen evolved was recorded with time at reaction temperatures of 30, 35, 40, and 45 °C. Other experimental parameters, such as the catalyst weight, NaBH_4_ concentration, base addition, and recycling, have been examined too at 35 °C.

The hydrogen generation rates (HGR) were calculated utilizing this equation:

3 $$\:\mathrm{H}\mathrm{G}\mathrm{R}\:(\mathrm{m}\mathrm{l}.{min}^{-1}.{g}^{-1})=\frac{{V}_{{H}_{2}\:}\left(ml\right)}{t\:\left(\mathrm{min})\:\mathrm{*}\:Wt.cat.\:(g\right)}$$

Additionally, this setup was also used for the estimation of OER from H_2_O_2_ decomposition. The evolved oxygen gas during H_2_O_2_ decomposition was measured by the water displacement from burettes. The catalytic run includes 10 mg of the prepared catalyst and 20 ml of H_2_O_2_ solution (30%, w/v), which were added to the vessel and connected to the glass apparatus. The evolved oxygen volume was recorded with reaction time at reaction temperatures of 30, 35, 40, and 45 °C. From analyzing the experimental data, the decomposition of H_2_O_2_ undergoes a first-order process.

The rate constant of this reaction has been calculated through non-linear fitting of a first-order reaction:

4 $$\:\mathrm{x}=\mathrm{a}(1-{\mathrm{e}}^{-\mathrm{k}\mathrm{t}})$$

Calculating the activation energy for the catalytic reactions with green-synthesized Co_3_O_4_ NPs by utilizing the Arrhenius equation as follows:

5 $$\:ln\left(k\right)=ln\left(A\right)-\frac{{\mathrm{E}}_{\mathrm{a}}}{\mathrm{R}\mathrm{T}}$$

In the Arrhenius equation, (k) is the rate constant of the reaction, (A) is a frequency factor, (E_a_) is an activation energy, (R) is a gas constant, and (T) is an absolute temperature in Kelvin.

## Result and discussion

### Characterization of Co_3_O_4_ NPs

**Fig. 2:**
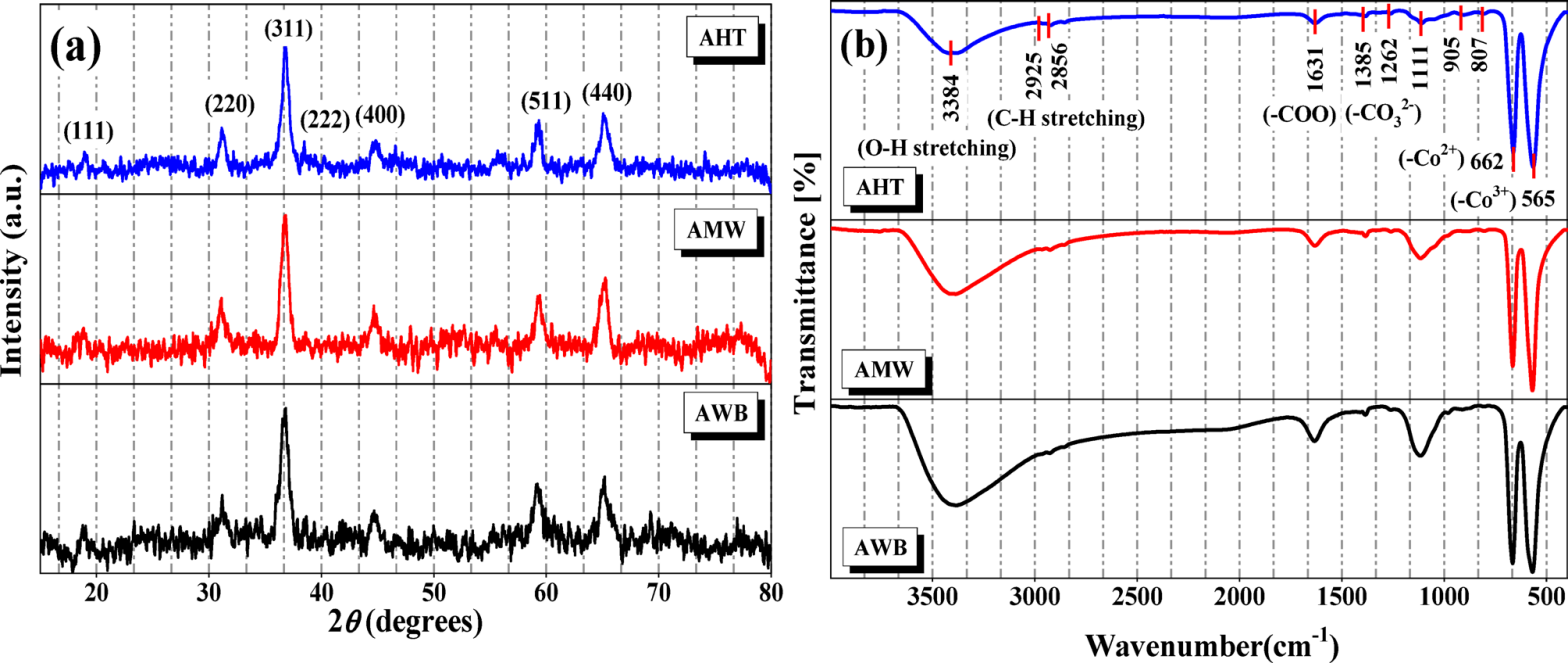
XRD pattern of AHT, AMW and AWB catalysts **(a)** and FT-IR spectra of the various synthesized Co_3_O_4_ NPs **(b)**.

**Fig. 3:**
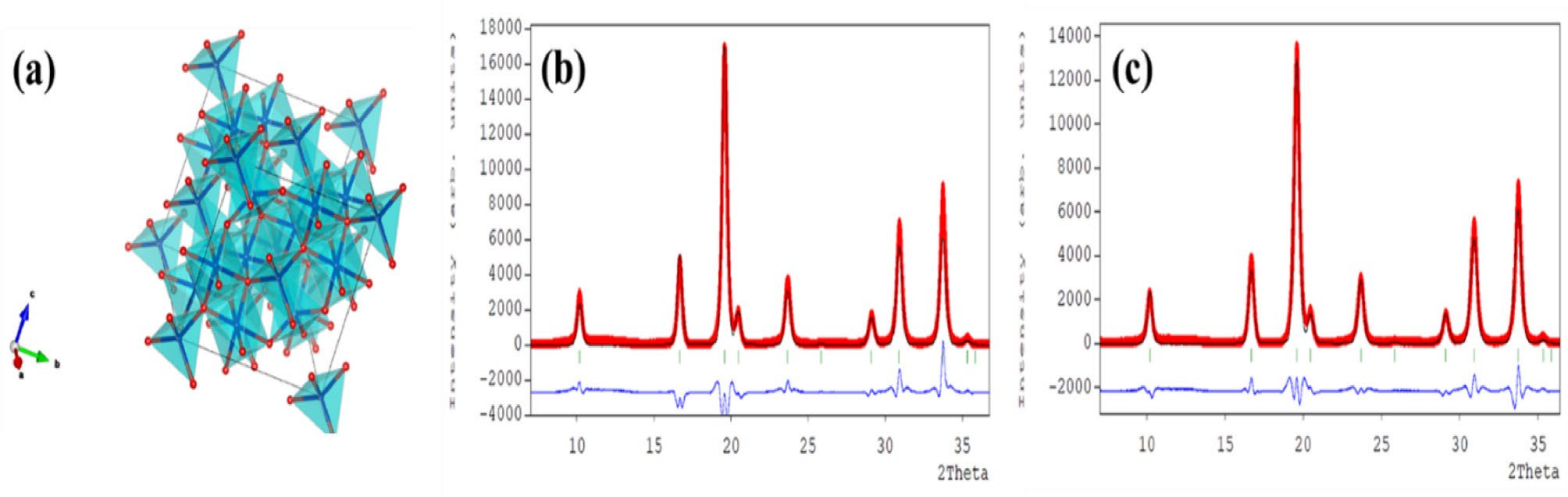
The chemical structure of Co_3_O_4_ NPs utilizing refinement data by Vesta software **(a)**, Refinement data of XRD pattern from SESAME synchrotron by Fullprof software: AMW **(b)** and AWB **(c)**.

Figure 2(a) reveals the XRD patterns of Co_3_O_4_ NPs synthesized by various methods utilizing green fuel (Aloe Vera leaves). XRD analysis confirmed the structural characteristics and demonstrated the presence of face-centered cubic phases and the crystallinity of the generated Co_3_O_4_ NPs. The characteristic peaks of Co_3_O_4_ NPs appeared at eight peaks, which are indexed to the planes (111), (220), (311), (222), (400), (422), (511), and (440). In the XRD patterns of Co_3_O_4_ NPs, the 2*θ* positions and FWHM of all diffraction peaks are well matched in accordance with the standard JCPDS (card No. 03–065-3103), which confirms the formation of Co_3_O_4_ NPs with higher purity. Additionally, the highest intensity peak was displayed at approximately 37°, which is related to their higher crystallinity and lattice strain. Almost all reflections from catalysts demonstrate approximately the same values of 2*θ* and d-spacing. Furthermore, the Scherrer equation was estimated to determine the average crystallite size of Co_3_O_4_ NPs (Eq. 6).

6 $$\:\mathrm{D}=\:\frac{\mathrm{k}\:{\uplambda\:}}{{\upbeta\:}\mathrm{cos}{\uptheta\:}}$$

where D is grain size, k = 0.9 is the Scherrer constant, λ is the X-ray wavelength, β is the FWHM (in radians), and *θ* is the diffraction angle. The average crystallite size of all catalysts was determined as 15.77, 17.3, and 14.03 nm, corresponding to AHT, AMW, and AWB, respectively. The diffractogram of synthesized catalysts verified the presence of Co_3_O_4_ spinel structure as the only phase without any other impurities from CoO and Co_2_O_3_. The XRD analysis revealed all patterns of Co_3_O_4_, without any indication of carbon species, which were detected in the XPS analysis (*vide infra*). The Synchrotron XRD patterns and measurement data for AMW and AWB catalysts are displayed in Fig. 3(b) and (c). The identical high-resolution XRD pattern of Co_3_O_4_ NPs in both catalysts confirms the presence of eight lattice planes, although these may be less visible in standard lab-scale XRD measurements. The catalysts’ XRD patterns align with the JCPDS card no. 96–900-5897. Using Rietveld refinement of Synchrotron XRD data with FullProf software^37^, atomic parameters and occupancy of oxygen and cobalt atoms are listed in Table 1. The catalysts possess a cubic crystal structure with space group Fd3m; the lattice parameters (a = b = c) are 8.08 Å for AMW and 7.74 Å for AWB. Additionally, the face-centered cubic structure of Co_3_O_4_ NPs depicted in Fig. 3(a) was generated with VESTA software^38^, based on the Rietveld refinement results.

**Table 1:** Atomic Parameters from Rietveld refinement of catalysts (AMW and AWB) data [SESAME, ID09 MS] (space group: Cubic, 3̅, wavelength (λ) = 0.83 Å, and JCPDS card number: 96–900-5897).

	AMW				AWB			
	X	Y	Z	Occ.	X	Y	Z	Occ.
Co^2+^	0.125	0.125	0.125	0.06937	0.125	0.125	0.125	0.04167
Co^3+^	0.5	0.5	0.5	016147	0.5	0.5	0.5	0.08333
O	0.26037	0.26037	0.26037	0.30452	0.2632	0.2632	0.2632	0.16667
Lattice parameter a=b=c (Å)	8.0837				7.742553			

FT-IR spectra verified the chemical composition of green-synthesized Co_3_O_4_ NPs. The spectra of the formed Co_3_O_4_ catalysts demonstrate functional groups at wavelength ranges between 4000 and 400 cm^− 1^, as depicted in Fig. 2(b). Almost all the absorption peaks of catalysts are approximately at the same wavenumber. By comparing the FT-IR spectra, the weak peak at 3759 cm^− 1^ may be related to the Co-OH vibration corresponding to the AMW catalyst; this band causes a decline in the catalytic performance (*vide infra*). The distinct sharp and broad absorption peaks at 3418–3384 cm^− 1^ indicate O-H stretching vibrations that could be indexed to adsorbed water molecules^6,39^. Moreover, there are two weak peaks located at around 2928–2925 cm^− 1^ and 2859 − 2856 cm^− 1^ that could be related to the symmetric and asymmetric C-H stretching^6,39^. These findings indicate the presence of carbonaceous surface species from the calcination residue of the green source. The signal observed at 1633–1631 cm^− 1^ could be related to the carboxylic groups and/or the bending vibration of H_2_O^6,39,40^. Furthermore, the surface carbonate vibrations were detected at two peaks around 1386–1384 cm^− 1^ and 1116 − 1111 cm^-1^^6,41^. The two peaks of AHT are located at approximately 905 cm^− 1^ and 807 cm^− 1^, corresponding to an aromatic C-H out-of-plane bending. Overall, the presence of various carbon species seems to have a small concentration, which could be assessed by XPS analysis (*vide infra*). The spinel structure of synthesized Co_3_O_4_ catalysts was confirmed by the presence of two strong peaks at 666–662 cm^− 1^ and 569 − 565 cm^− 1^, corresponding to the cobalt-oxygen stretching vibration. The peak at approximately 666 cm^− 1^ is assigned to Co^2+^ vibrations at a tetrahedral site. Correspondingly, the peak at around 565 cm^− 1^ is ascribed to Co^3+^ vibrations within an octahedral site of the spinel Co_3_O_4_ framework^1,6,8^. The XRD and FT-IR results are straightforward evidence for the enhancement of biosynthesis of Co_3_O_4_ NPs.

**Fig. 4:**
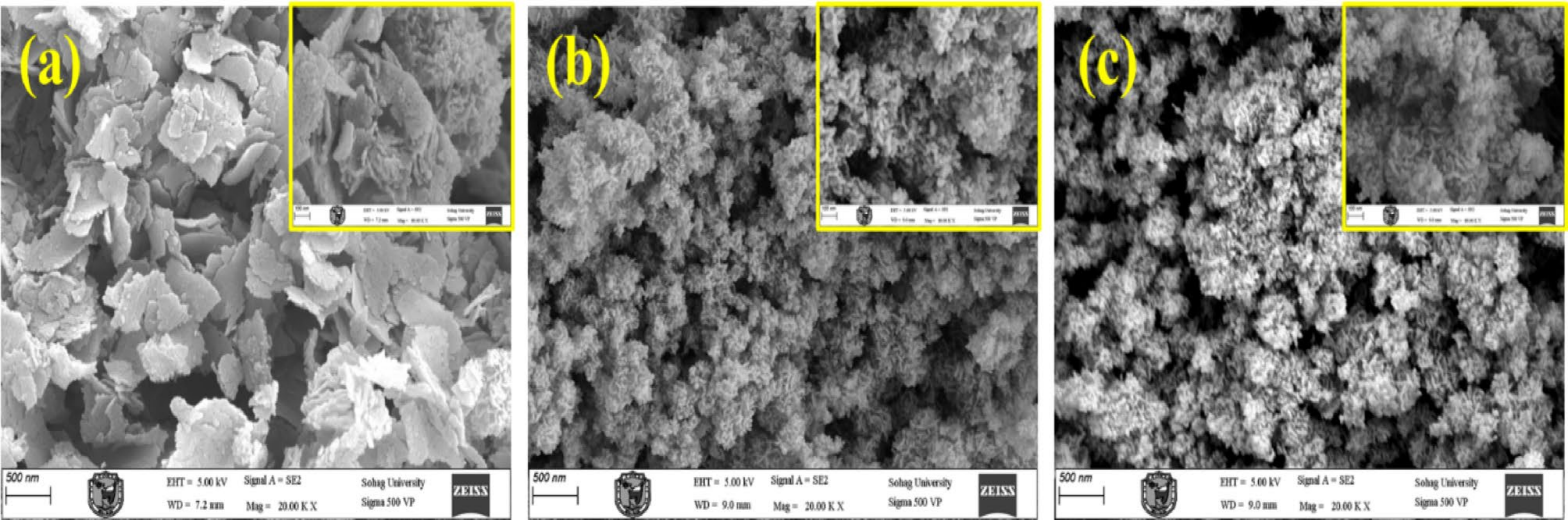
FESEM images of the formed Co_3_O_4_ NPs by various techniques: AHT **(a)**, AMW **(b)**, AWB **(c)**.

The FESEM characterization was used to determine the external-surface morphology of catalysts. As seen in Fig. 4(a), FESEM images of the AHT catalyst show a distinct flower-like and sheet-like morphology. Some particles exhibit a spherical shape with agglomeration. Additionally, the FESEM images of the AHT catalyst reveal numerous pores and voids, which are the primary reason for the higher catalytic performance in NaBH_4_ hydrolysis. These nanographs of Co_3_O_4_ NPs display characteristics of well-agglomerated particles alongside observable voids and pores due to the reduction of surface area and the Ostwald ripening phenomenon. Other catalysts (AMW and AWB) exhibit a similar morphology characterized by the agglomeration of spherical particles in the network, as shown in Fig. 4(b) and (c). The NPs of these catalysts demonstrate a uniform capsule-like shape with notable agglomeration.

N_2_ physisorption measurements were utilized to examine the textural properties of the prepared samples. The N_2_ adsorption-desorption isotherms and pore size distribution patterns by the DFT method are illustrated in Fig. S1. The catalysts displayed a mixture of Type II and Type IV isotherms and a hysteresis loop of type H3 according to the IUPAC classification. At high relative pressures, the adsorbed volume increases with the thickness of the adsorbed layer until P/P_o_ = 1. The hysteresis loop type H3 appears at P/P_o_ = 0.7, indicating the presence of plate-like particles forming slit-like pores (Fig. S1(a)). The pore volumes of catalysts were measured by the BJH method, whereby the AHT catalyst exhibited a lower pore volume, which impacts its catalytic performance. The BET method was used to determine the computed specific surface area (S_BET_) of the synthesized catalysts, which were found to be 57, 96, and 116 m^2^g^− 1^, corresponding to AHT, AMW, and AWB. These values are larger than reported for combustion synthesis of Co_3_O_4_ NPs calcined at 400 °C with 39.5 m^2^g^-1^^10^. Chen, Huihui, et al. reported that two prepared catalysts (Co_3_O_4_-air and Co_3_O_4_-H_2_O_2_) are 81.3 and 79.8 m^2^g^-1^^35^, respectively, which are lower than the values of AMW and AWB. Abu-Zied et al. reported that green synthesis of Co_3_O_4_/C nanocomposite employing basil leaves extract calcined at 400 °C with a lower surface area of 19 m^2^g^-1^^1^. Moreover, these values are extremely higher than those reported for the green-synthesized Co_3_O_4_ NPs using jasmine flowers extract and subsequent calcination at 300 °C (6 m^2^g^− 1^) and 500 °C (2 m^2^g^− 1^)^6^. These results highlight the crucial contribution of the green precursor in controlling the texture of the synthesized nanomaterials. The total N_2_ volume absorbed for the AWB catalyst is approximately 123 cm³g^− 1^, while the maximum volume absorbed is observed for the AMW and AHT catalysts, with values of 108 and 68 cm³g^− 1^, respectively. The particle size distribution was plotted as shown in Fig. S1(b) employing the DFT method. Inspection of the obtained size distribution of the formed catalysts (AHT and AMW) reveals the existence of poly-disperse character and three peaks at different regions. Most of the NPs lie in the mesoporous range, with sharp peaks consisting of two maxima at pore widths of 9.9 and 29.6 nm. The other board peak can be observed at a pore width of approximately 79.8 nm, which is in the vicinity of the macroporous region. Additionally, a peak was observed only in AHT at a pore width of 3.9 nm. Comparing the distribution size of the synthesized catalysts, the AWB catalyst shows two peaks in the mesoporous region at 6.5 and 29.6 nm, as shown in Fig. S1(b). All texture properties of the prepared catalysts are listed in Table 2, including the BET surface area, pore volume, and pore size.

**Table 2:** Textural data is abstracted from the N_2_ adsorption data of AHT, AMW, and AWB.

Catalyst	BET surface area (m^2^/g)	BJH pore volume (cc/g)	BJH pore size (nm)
**AHT**	57	0.078	1.75
**AMW**	96	0.124	1.73
**AWB**	116	0.145	1.76

**Fig. 5:**
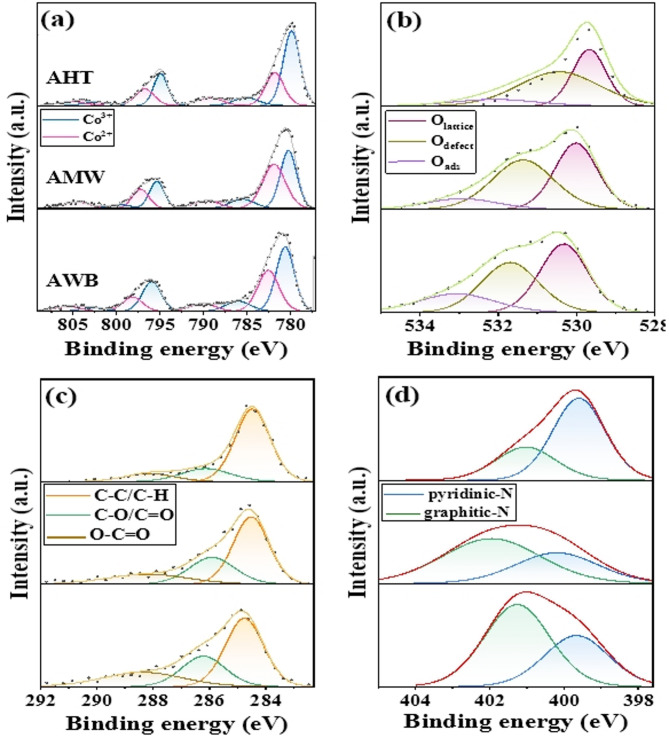
XPS data obtained from the prepared catalysts (AHT, AMW, and AWB): Co 2p **(a)**, O 1 s **(b)**, C 1 s **(c)**, and N 1 s **(d)** fitted spectra.

The elemental composition of the green-synthesized Co_3_O_4_ NPs was further examined by XPS analysis. The XPS survey spectra of Co_3_O_4_ NPs (Fig. S2) indicate the coexistence of Co, O, C, and N atoms on the surface of the catalysts, which reflects the higher purity of the samples. From the survey spectra, the presence of C and N atoms is attributed to the phytochemicals in the aloe vera extract that adhere to the surface of Co_3_O_4_ nanoparticles, functioning as stabilizing agents. Table 3 shows an increasing atomic concentration of Co 2p in the survey, as ordered AHT > AWB > AMW. The higher percentage causes an increase in the active sites, which enhances the catalytic performance of NaBH_4_. The lower atomic concentration of N in the AHT catalyst may be responsible for superior catalytic efficiency. The Co 2p core spectra of all catalysts (Fig. 5(a)) demonstrate two main peaks at BE values of 780.38-779.58 eV and 781.97-780.75 eV, corresponding to Co 2p_3/2_ and Co 2p_1/2_ spin-orbit peaks, respectively^4,6,42^. The first two peaks could be associated with the presence of Co^3+^ and Co^2+^ ions at the surface, corresponding to the deconvolution of Co 2p_3/2_. Similarly, four Co 2p_3/2_ peaks of all catalysts can be observed at BEs of 780.38-779.58 eV, 781.97-780.75 eV, 585.98-585.07, and 795.53-794.65 eV, corresponding to Co^3+^, Co^2+^, and satellites, respectively. The two asymmetric broad peaks were characteristic of Co 2p_1/2_ and Co 2p_3/2,_with spin-orbit splitting of approximately 15 eV. In agreement, Junshan Han et al.^43^ detected the same BE values of Co_3_O_4_ PNS sample as in AHT sample at 779.7 eV and 780.9 eV of Co 2p_3/2_ spin state attributable to Co^3+^ and Co^2+^, which verifies the presence of Co^3+^ and Co^2+^ oxidation states of Co_3_O_4_ NPs. Abu-Zied et al.^1^ reported in XPS spectra that the presence of the Co(OH)_2_ peak at the BE 789.55 eV. For instance, this peak appeared in the XPS spectrum of AMW, approximately at 790.07 eV, and the FT-IR spectrum of AMW revealed the existence of Co(OH)_2_. From O 1 s spectra, three peaks are found in all samples, which are assigned to lattice oxygen (O^2−^), oxygen defect (OH^−^), and surface oxygen (H_2_O)^4,42^. The O1s spectrum of AHT (Fig. 5(b)) displayed three peaks at 529.67 eV, 530.45 eV, and 532.05 eV, respectively. Other catalysts have the same peaks with BEs shift of ± 0.5 eV. The superior performance observed in AHT is due to its higher ratio of Co^2+^/Co^3+^ and the lower ratio of oxygen vacancies (O_lattice_/O_defect_ (%)) compared to AMW and AWB, as detailed in Table 4. The high-resolution scan of C1s spectra in AHT (Fig. 5(c)) exhibited three peaks at 284.47 eV (C-C/C-H)^24^, 286.04 eV (C-O/C = O)^6,24^, and 287.34 eV (O-C = O)^6,15,44^. The other catalysts have similarities in peaks approximately at the same BE. These findings confirmed the carbonaceous species observed in IR spectra, and the lower (C-O/C = O) content in AHT resulted in higher activity during NaBH_4_ hydrolysis. The N 1 s spectrum of AHT (Fig. 5(d)) is well fitted into two small peaks at BE 399.16 and 400.71 eV, attributable to pyridinic-N and graphitic-N, respectively, as mentioned in the literature^45^. Moreover, similar peaks have been detected at the surface of AWB and AMW catalysts. The presence of nitrogen in all samples may be considered as the residue of the green synthesis by aloe vera plants.

**Table 3:** XPS surface binding energies and atomic percentages of the elements detected in the survey of synthesized catalysts.

Element	AHT		AMW		AWB	
	BE (eV)	Atomic (%)	BE (eV)	Atomic (%)	BE (eV)	Atomic (%)
**Co 2p**	781.16	30.91	781.74	28.68	782.08	30.65
**O 1s**	531.06	49.97	531.53	50.46	532.06	50.63
**C 1s**	285.51	18.6	286.01	19.82	286.22	17.61
**N 1s**	401.12	0.52	401.39	1.04	402.1	1.11

**Table 4:** XPS binding energy and atomic percentage of Co2p, O1s, C 1 s and N1s for the prepared catalysts AHT, AMW and AWB.

Catalyst	AHT		AMW		AWB	
	BE (eV)	Atomic (%)	BE (eV)	Atomic (%)	BE (eV)	Atomic (%)
Co^3+^ (2p_3/2_) (2p_1/2_)	779.58 794.65	13.82 6.61	780.03 795	19.41 5.31	780.38 795.53	19.27 7.84
Co^2+^ (2p_3/2_) (2p_1/2_)	780.75 796.1	43.41 17.87	781.52 796.49	37.48 17.23	781.97 797.23	37.89 16.07
Ratio of Co^2+^/Co^3+^ (2p_3/2_)	3.14		1.93		1.97	
O 1 s (lattice oxygen)	529.67	36.37	530.01	42.59	530.32	43.18
O 1 s (oxygen defect)	530.45	52.62	531.37	45.83	531.7	37.76
O 1 s (surface oxygen (H_2_O))	532.08	11.01	532.94	11.58	533.07	19.06
Ratio of O_lattice_/O_defect_	0.69		0.93		1.14	
C 1 s (C-C/C-H)	284.47	72.82	284.52	54.24	284.76	51.12
C 1 s (C-O/C=O)	286.04	5.13	285.91	27.49	286.22	26.12
C 1 s (O-C=O)	287.34	22.05	288.2	18.28	288.37	22.76
N 1 s (pyridinic-N)	399.16	68.3	399.81	36.71	399.75	1.92
N 1 s (graphitic-N)	400.71	31.7	401.7	63.29	400.75	98.08

### Catalytic hydrogen generation via NaBH_4_ hydrolysis

**Fig. 6:**
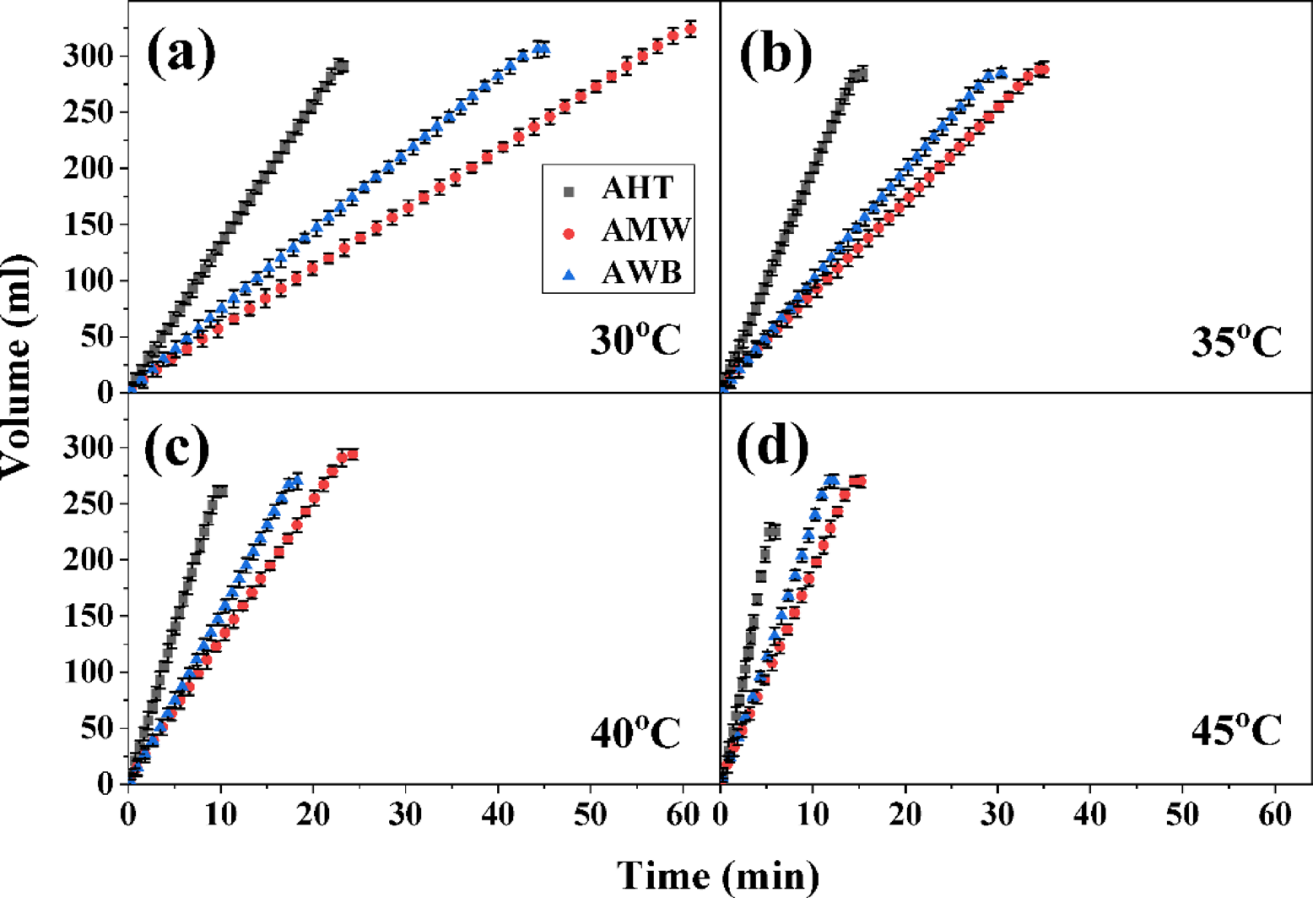
Abstracted data from V_H2_-time curves during NaBH_4_ hydrolysis over Co_3_O_4_ NPs at different reaction temperatures: 30 °C **(a)**, 35 °C **(b)**, 40 °C **(c)**, 45 °C **(d)**. The conditions remain consistent with 10 mg of synthesized catalyst and 20 ml of 0.75 wt% NaBH_4_.

**Fig. 7:**
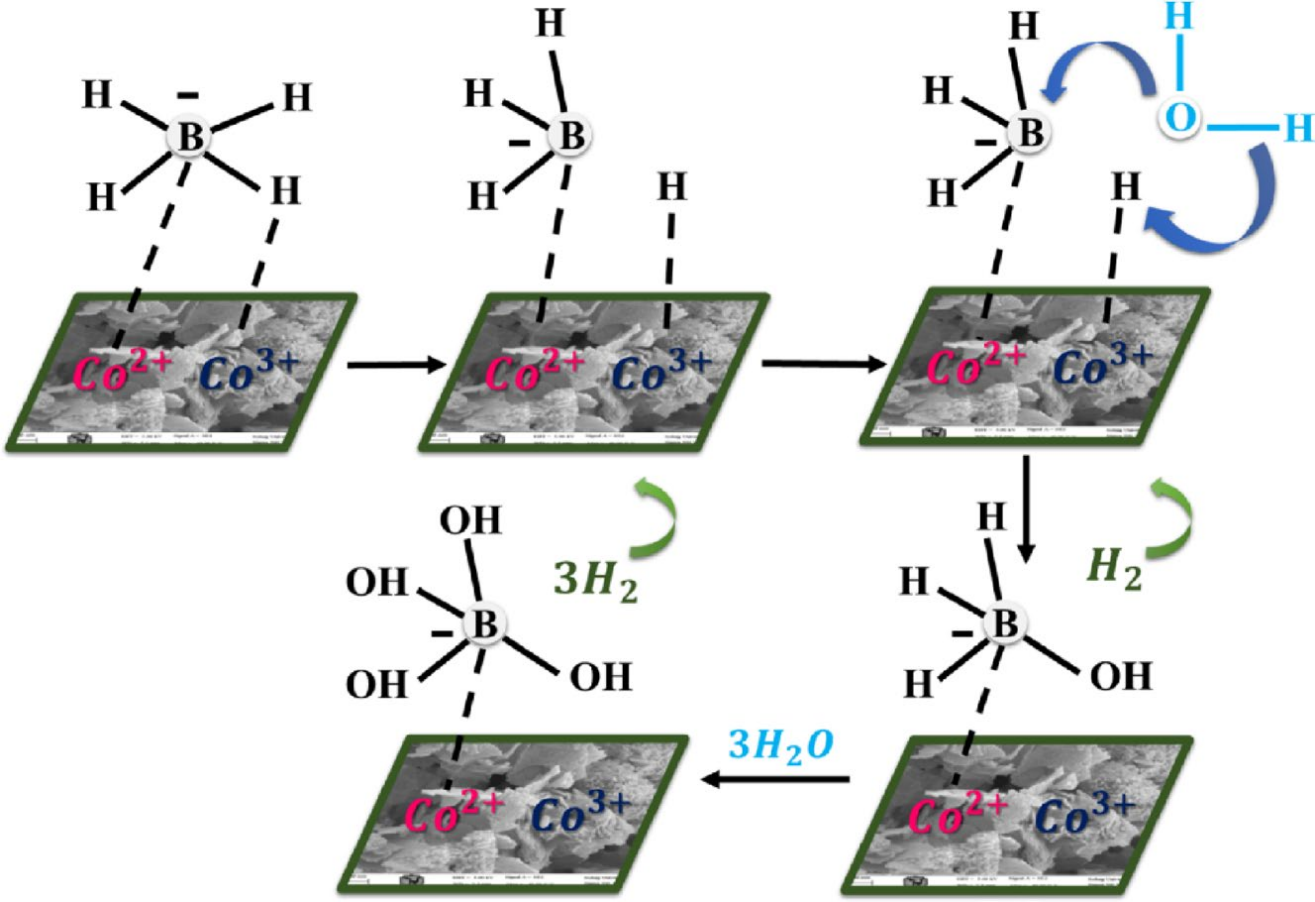
Supposed mechanism of the catalytic hydrolysis of NaBH_4_ by green-synthesized Co_3_O_4_ NPs.

The catalytic activity was measured at various reaction temperatures (30–45 °C) for the obtained catalysts using 10 mg of each catalyst and 20 ml of NaBH_4_ solution (0.75% wt.). As depicted in Fig. 6, any plot shows that the volume of evolved hydrogen was recorded as a function of time. The volume of released hydrogen at a lower temperature (30 °C) after 20 min was 258, 144, and 111 ml for AHT, AWB, and AMW, respectively. These plots demonstrate linearity with a gradual increase in catalytic activity as reaction temperatures elevate. The values of HGR were computed by using linear fitting of these plots during the hydrolysis of NaBH_4_ over the catalysts. To evaluate the catalytic performance of the synthesized catalysts, HGR values were calculated at 30 °C, yielding 1271, 696, and 530 ml.min^− 1^.g^− 1^, corresponding to AHT, AWB, and AMW, respectively. AHT catalyst exhibits the highest HGR value among all synthesized catalysts, while the other catalysts (AMW and AWB) have similarly close HGR values, as shown in Fig. S3. The HGR values of the optimal catalyst (AHT) are 1271, 1954, 2715, and 4267 ml.min^− 1^.g^− 1^. This data is significantly higher compared to the HGR values of other catalysts listed in Table 5. Additionally, the activation energies (E_a_) of all catalysts were calculated by using the Arrhenius plot. Whereas the Arrhenius plots are the relationship between ln (k) and 1/T, as illustrated in Fig. S4. The recorded values of E_a_ are 63.62, 65.69, and 68.19 kJ/mol for AHT, AMW, and AWB, respectively. These findings confirmed that the value of E_a_ decreased with the higher catalytic performance reaction. The lower activation energy is directly related to enhanced surface defects (fewer oxygen vacancies) and the greater accessibility of Co^2+^ (higher content), which favors electronic interactions within the catalyst structure that promote the cleavage of B-H bonds and accelerate the hydrolysis of NaBH_4_. The catalytic hydrolysis of the NaBH_4_ reaction has two main proposed mechanisms, either the Eley-Rideal or Langmuir-Hinshelwood mechanism. Firstly, in the Eley-Rideal mechanism (Fig. 7), only one reactant (BH_4_^–^) adsorbs on the catalyst surface. One of the hydrogen atoms migrates from (BH_4_^–^) to the unoccupied active center. Also, the other reactant (H_2_O) reacts with it directly. The hydrogen gas was obtained from combining two hydrogen atoms from the borohydride anion and a water molecule. Then, the hydroxyl ion from H_2_O reacts with BH_3_ and forms BH_3_(OH)^–^. After that, these steps repeat to give three H_2_ molecules and B(OH)_4_^–^ as a byproduct. This mechanism depends on the availability of unoccupied active sites at the catalyst surface. Whereas the Langmuir-Hinshelwood mechanism was explained step-by-step in several literatures^1,6,46^ reported that adsorption of BH_4_^–^ ions and H_2_O molecules on the Co_3_O_4_ surface. Co_3_O_4_ provides active sites for the nucleophilic attack and electron transfer from BH_4_^–^ ions. This leads to cleavage of the B–H bond, released hydrogen interacts with a proton source of water molecule (H^+^) and forms H_2_ gas. Each BH_4_^–^ ion can release four hydrogen atoms; hydrogen generation continues through successive hydrolysis steps involving intermediates:

$$\:{\mathrm{B}\mathrm{H}}_{3}{\left(\mathrm{O}\mathrm{H}\right)}^{-}\to\:\:\mathrm{B}{\mathrm{H}}_{2}(\mathrm{O}\mathrm{H}{)}_{2}^{-}\to\:\:\mathrm{B}\mathrm{H}(\mathrm{O}\mathrm{H}{)}_{3}^{-}\to\:\mathrm{B}({\mathrm{O}\mathrm{H})}_{4}^{-}$$

**Table 5:** Comparison of HGR values of various catalysts utilized for NaBH_4_ hydrolysis.

Catalyst	Synthesis approach	Conditions	E_a_ (kJ/mol)	HGR (ml.min^−1^.g^−1^)	Ref.
Co/MWCNTs-30	Impregnation/annealing	1 wt% NaBH_4_, 35 °C	53.8	295	^46^
Cobalt sulphide	Hydrothermal technique	6.4 wt% NaBH_4,_ 25 °C	58.8	328	^56^
Co_3_O_4_	Microwave irradiation method	0.75 wt% NaBH_4_, 30 °C	81.57	412	^4^
Co_3_O_4_	Commercial	10 wt% NaBH_4_, 8 wt% NaOH, 25 °C	77.9	860	^57^
Co-basil-500	Basil leaves extract	1 wt% NaBH_4_, 25 °C	52.4	993	^1^
Co/Co_3_O_4_	Microwave irradiation-polyol method	10 wt% NaBH_4_, 10 wt% NaOH, 25 °C	-------	2823	^58^
AMW	Microwave irradiation method	0.75 wt% NaBH_4_, 30 °C	65.69	518	This work
AWB	Co-precipitation method	0.75 wt% NaBH_4_, 30 °C	68.19	696	This work
AHT	Hydrothermal method	0.75 wt% NaBH_4_, 30 °C	63.62	1271	This work

Significant investigations have been conducted on the kinetics of the hydrolysis reaction to understand the behavior of both the catalyst and reactants. Therefore, the hydrolysis reaction catalyzed by AHT at various temperatures is examined through two kinetic models: zero-order and Langmuir-Hinshelwood.

The plots of NaBH_4_ hydrolysis using AHT exhibited a straight-line behavior; this suggests that the reaction undergoes zero-order kinetics^47,48^, which can be determined by utilizing mathematical equations, as follows:

7 $$\:{\mathrm{r}}_{{\mathrm{N}\mathrm{a}\mathrm{B}\mathrm{H}}_{4}}=\:-\frac{\mathrm{d}{\mathrm{C}}_{\mathrm{t}}}{\mathrm{d}\mathrm{t}}=\mathrm{k}\left(\mathrm{t}\right)$$

Through the integration method of the differential Eq. (7), the resultant Eq. (8) is obtained as follows:

8 $$\:{\mathrm{C}}_{\mathrm{o}}\:-\:{\mathrm{C}}_{\mathrm{t}}\:=\mathrm{k}\mathrm{t}$$

Where C_o_ is the initial concentration of NaBH_4_, C_t_ is the concentration of NaBH_4_ at any time t, r is the reaction rate, and k is the reaction rate constant based on the solution volume. As shown in Fig. S5, plotting of C_t_ versus time produces a straight line passing through the origin; its slope allows calculation of the zero-order rate constant (k), as listed in Table S1.

Among various models used to analyze the experimental data from NaBH_4_ hydrolysis, the Langmuir-Hinshelwood kinetic model^48^ was employed to elucidate the reaction mechanism.

The rate expression corresponding to this model can be expressed as follows:

9 $$\:-\:{\mathrm{r}}_{\mathrm{N}\mathrm{a}\mathrm{B}{\mathrm{H}}_{4}\:}=\:-\:\frac{{\mathrm{d}\mathrm{C}}_{\mathrm{t}}}{\mathrm{d}\mathrm{t}}=\mathrm{k}\:\frac{{\mathrm{K}}_{\mathrm{a}\:}{\mathrm{C}}_{\mathrm{t}}}{1+\:{\mathrm{K}}_{\mathrm{a}\:}{\mathrm{C}}_{\mathrm{t}}}$$

By integrating this Eq. (10), Eq. (11) was obtained

10 $$\:\frac{1}{{\mathrm{K}}_{\mathrm{a}}}\mathrm{ln}\left(\frac{{\mathrm{C}}_{\mathrm{o}}}{{\mathrm{C}}_{\mathrm{t}}}\right)+\left({\mathrm{C}}_{\mathrm{o}}-\:{\mathrm{C}}_{\mathrm{t}}\right)=\mathrm{k}\mathrm{t}$$

11 $$\:\mathrm{C}\:\left(\mathrm{t}\right)=\:\frac{1}{{\mathrm{K}}_{\mathrm{a}}}\:\mathrm{W}\:\left({\mathrm{K}}_{\mathrm{a}}{\mathrm{C}}_{\mathrm{o}}{\mathrm{e}}^{{\mathrm{K}}_{\mathrm{a}}{(\mathrm{C}}_{\mathrm{o}}-\mathrm{k}\mathrm{t})}\right)$$

Its rate expression is determined in this model by two constants: k (rate constant) and $\:{\mathrm{K}}_{\mathrm{a}}$ (adsorption constant). The Lambert W function^49^(Eq. (11)) is used with OriginPro software to determine these constants for the AHT catalyst at different temperatures, as depicted in Fig. S6 and listed in Table S2.

Additional experiments have been performed to evaluate the effect of parameters (catalyst weight, NaBH_4_ concentration, alkalinity, and recycling) on the catalytic activity of AHT.

**Fig. 8:**
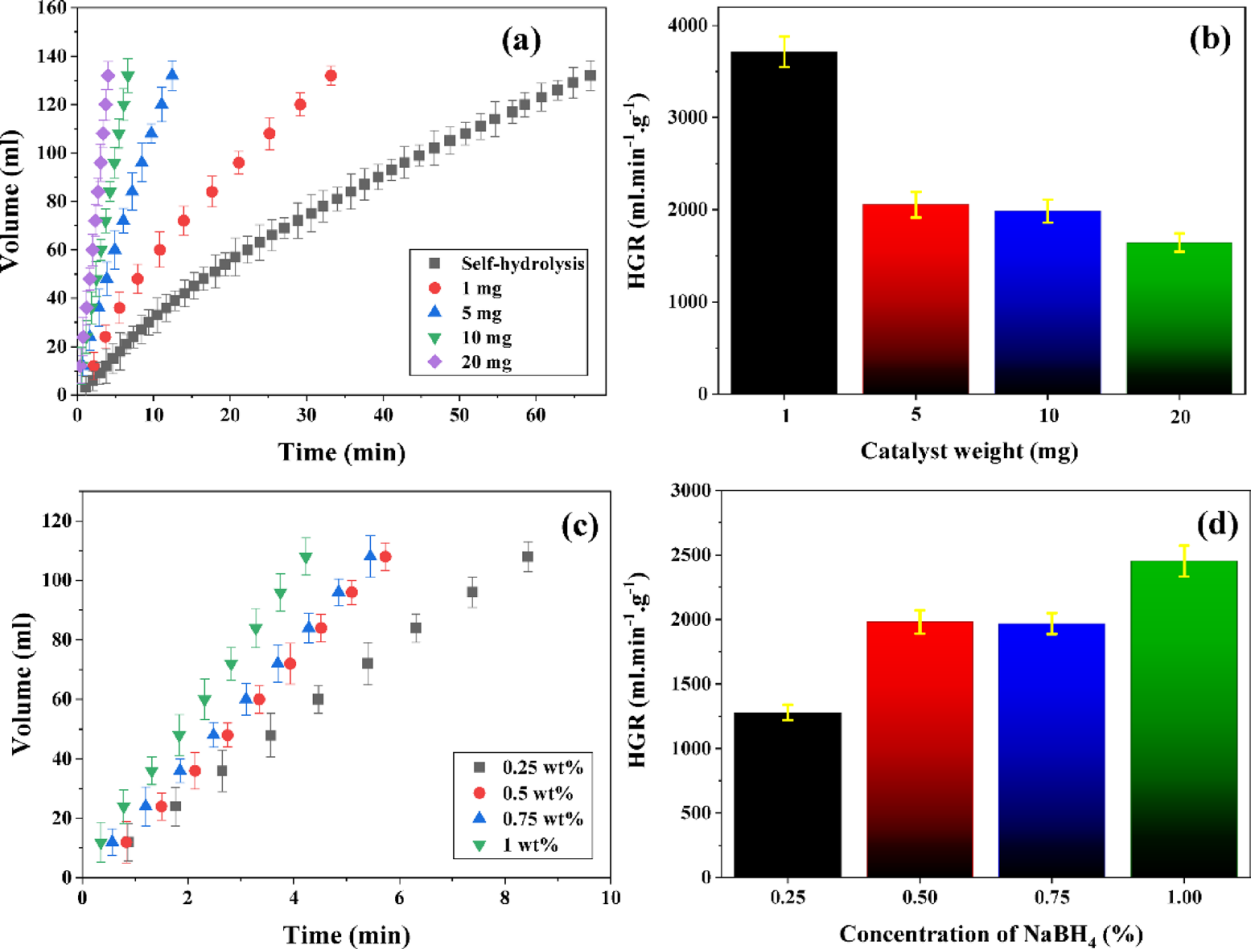
Effect of catalyst weight with various amounts of 1, 5, 10, and 20 mg of AHT using 20 ml of 0.75 wt% NaBH_4_ at 35 °C: V_H2_-time curves **(a)** and HGR vs. catalyst weight **(b)**. Effect of NaBH_4_ concentration with varying concentrations of 0.25, 0.5, 0.75, and 1 wt% using 10 mg of AHT at 35 °C: (V_H2_-time curves **(c)** and HGR vs. NaBH_4_ concentration **(d)** on hydrogen production over Co_3_O_4_ NPs.

In analyzing the impact of catalyst dosage on the hydrolysis of NaBH_4_ reaction, as seen in Fig. 8(a). Different amounts of AHT were examined: 1 mg, 5 mg, 10 mg, and 20 mg, using 150 mg of NaBH_4_ at 35 °C. Self-hydrolysis of NaBH_4_ was measured in the absence of a catalyst to evaluate the effect of the catalyst on HGR. The addition of 1 mg of AHT to the hydrolysis reaction obviously shows acceleration of the reaction in 21.13 min compared to self-hydrolysis in 42.82 min at 96 ml of hydrogen gas. Hence, the rate constant (k) value is approximately double in a small amount of catalyst (1 mg) than in self-hydrolysis. The value of HGR in 1 mg of catalyst weight is 3714 ml.min^− 1^.g^− 1^ compared to 1.9 ml.min^− 1^ in self-hydrolysis. These findings demonstrate the efficiency of the catalyst even in small amounts toward NaBH_4_ hydrolysis. By varying the catalyst amount from 1 to 20 mg at 35 °C, the HGR value (Fig. 8(b)) declined from 3714 to 1644 ml.min^− 1^.g^− 1^. The volume of hydrogen production was 132 ml in 33.18 min under the reaction conditions (1 mg Co_3_O_4_ NPs and 150 mg NaBH_4_) at 35 °C, corresponding to the higher HGR of 3714 ml.min^− 1^.g^− 1^. Despite the smaller catalyst weight, HGRs are higher at lower amounts rather than higher amounts because the HGR value is calculated by dividing the k value by the amount of catalyst. This results in a higher HGR value with smaller catalyst weights. Additionally, the plot of ln k versus ln [catalyst weight] was linear as revealed in Fig. S7, with a slope of 0.78 and a strong fit, evidenced by an R^2^ value of 0.98 for these experiments. Hassan et al. and Beheshti et al. reported smaller slope values in this relationship^6,50^. The kinetic study confirmed that the hydrolysis reaction proceeds as a first-order reaction.

Furthermore, the influence of NaBH_4_ concentrations on catalytic hydrolysis and their reflection on HGR values was studied. The NaBH_4_ concentrations examined were 0.25, 0.5, 0.75, and 1 wt%, with a constant catalyst amount of 10 mg at 35 °C (Fig. 8(c)). Therefore, higher NaBH_4_ concentrations resulted in increased HGR values (Fig. 8(d)), from 1278 ml.min^− 1^.g^− 1^ at 0.25 wt% to 2452 ml.min^− 1^.g^− 1^ at 1 wt% NaBH_4_. 108 ml of H_2_ evolved in 8 min at 0.25 wt% NaBH_4_, whereas at the higher concentration of 1 wt% NaBH_4_, 108 ml of H_2_ was liberated in 4 min.

**Fig. 9:**
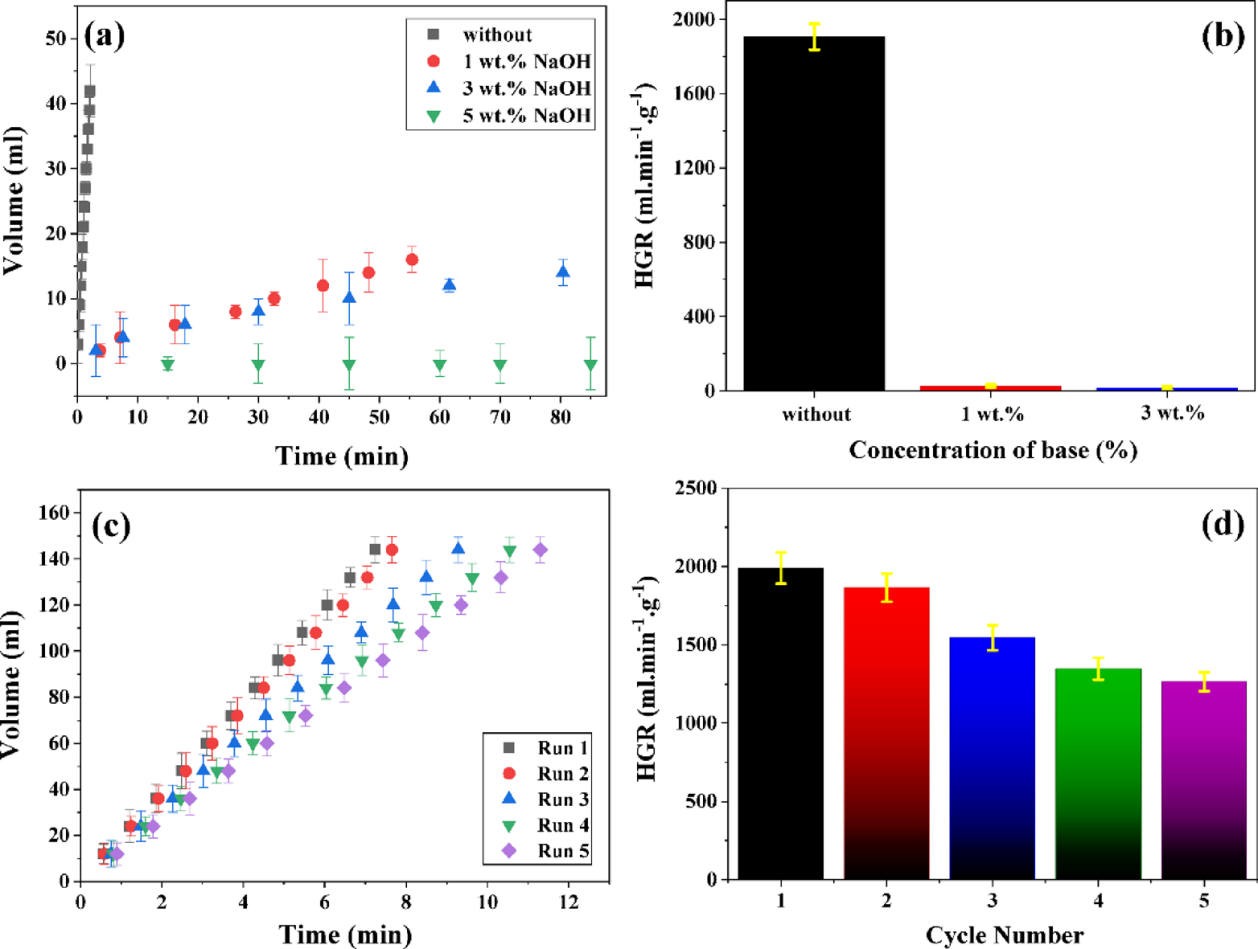
Effect of NaOH concentration across varying concentrations of 1, 3, and 5 wt% using 10 mg of AHT and 20 ml of 0.75 wt% NaBH_4_ at 35 °C: V_H2_-time curves **(a)** and HGR vs. base concentration **(b)**. Effect of catalyst recyclability on hydrogen generation over AHT catalyst under fixed conditions (10 mg of AHT and 20 ml of 0.75 wt% NaBH_4_ at 35 °C): V_H2_-time curves **(c)** and HGR vs. cycle number **(d)**.

To investigate the effect of alkalinity on H_2_ generation during NaBH_4_ hydrolysis, three different concentrations of NaOH were studied (Fig. 9(a)). To reduce the impact of spontaneous self-hydrolysis of NaBH_4_, an alkaline medium such as NaOH was used to ensure the stability of the solution during storage^21^. As shown in Fig. 9(b), HGR values of 26 and 15 ml.min^− 1^.g^− 1^, corresponded to 1 wt%, 3 wt%, and higher NaOH concentrations, respectively. Without NaOH as a basic medium, there was higher activity in the hydrolysis reaction, with an efficient HGR of 1906 ml.min^− 1^.g^− 1^. The HGR values decreased with increasing base concentration until reaching 5 wt%, where the activity diminished due to increased solution viscosity. The solubility of NaBO_2_ was decreased by increasing base concentrations (OH^−^ ions), which can hinder this reaction. This leads to a decline in catalytic activity by occupying active centers on the catalyst surface^22^. Demirci et al. illustrated the effect of increasing NaOH concentrations, which decreased the HGR in continuous activity^51^.

In industrial developments, it is crucial to determine the durability of catalysts after multiple runs. The synthesized AHT catalyst was tested for reusability by recycling it over 5 runs. In each run, the reaction used 10 mg of catalyst with 150 mg of NaBH_4_ dissolved in 20 ml of deionized water, and the time versus H_2_ volume was recorded (Fig. 9(c)). The catalyst was then collected by filtration, washed with distilled water to remove any NaBO_2_ buildup on the surface, and dried in air. The recycled catalyst was then used again with fresh NaBH_4_, repeating these steps in four additional runs. Results showed that the HGR values declined over the 5 runs, with the conversion from the first cycle to the last being 64%. Moreover, the HGR values (Fig. 9(d)) were obtained as 1990, 1865, 1546, 1347, and 1265 ml.min^− 1^.g^− 1^, respectively. A 5-cycle test was conducted to evaluate the sustainability of the catalyst during recycling and to assess degradation of catalyst activity after multiple runs. The reasons for catalyst performance degradation could include factors such as the reduction of active sites, changes in catalyst morphology, and the formation of inactive species on the surface. To verify the reduction in catalytic activity of the AHT catalyst, XRD analysis was conducted after the 1 st and 5th cycles to compare the obtained crystal planes before and after following the recycling process. Along with an increase in carbon species, as evidenced by the loss of planes and the higher presence of carbon content, this may contribute to a decline in catalytic performance with repeated use. FE-SEM analysis supported the XRD findings, revealing changes in the catalyst’s morphology after the 5th cycle. The catalyst’s structure altered from nanoflower and sheet forms to an agglomeration of spherical NPs, which appeared capsule-like with more buildup on the catalyst surface.

Overall, the recycling data indicate that the synthesized AHT catalyst maintains higher efficiency and stability despite multiple runs, with no degradation of catalyst activity after each run. The reasons for degradation of the catalyst performance could be caused by several factors, such as the reduction of active sites, the changing morphology of the catalyst, and the creation of inactive species on the surface. In essence, the collected recycling data indicate that the synthesized AHT catalyst has higher efficiency and stability despite several runs.

### Catalytic oxygen evolution via H_2_O_2_ decomposition

In the anticipated future, H_2_O_2_ and transition metal oxides will be important constituents of energy production processes^52^. Additionally, the H_2_O_2_ solution has an oxygen storage capacity approximately 1600 times greater than that of the atmosphere. The breakdown of H_2_O_2_ on catalysts is essential for fuel cells and medical science as a clean oxygen source and sustainable fuel. Therefore, a clean and highly efficient catalyst is crucial for producing pure oxygen and water through H_2_O_2_ decomposition. The catalytic performance of the synthesized catalysts was evaluated by H_2_O_2_ decomposition as a model reaction.

G´omez-Largo et al. have reported that the two possible reaction mechanisms of the decomposition of H_2_O_2_ catalyzed by transition metal oxides are as follows: (i) the surface oxygen vacancies mechanism^53,54^ and (ii) the radical mechanism^10,54^. In the first mechanism, a H_2_O_2_ molecule reacts with a surface oxygen vacancy (V_(surf)_) of the oxide to form an adsorbed oxygen species (O_(ads)_) (reaction 12), which subsequently desorbs as O_2_ (reaction 13).

12 $${{\rm{V}}_{({\rm{surf}})}} + {\rm{ }}{{\rm{H}}_{\rm{2}}}{{\rm{O}}_{\rm{2}}} \to {\rm{ }}{{\rm{V}}_{({\rm{surf}})}}{-}{\rm{ }}{{\rm{O}}_{({\rm{ads}})}} + {\rm{ }}{{\rm{H}}_{\rm{2}}}{\rm{O}}$$

13 $${{\rm{V}}_{({\rm{surf}})}} - {\rm{ }}{{\rm{O}}_{({\rm{ads}})}} + {\rm{ }}{{\rm{H}}_{\rm{2}}}{{\rm{O}}_{\rm{2}}} \to {\rm{ }}{{\rm{V}}_{({\rm{surf}})}} + {\rm{ }}{{\rm{H}}_{\rm{2}}}{\rm{O }} + {\rm{ }}{{\rm{O}}_{\rm{2}}}$$

**Fig. 10:**
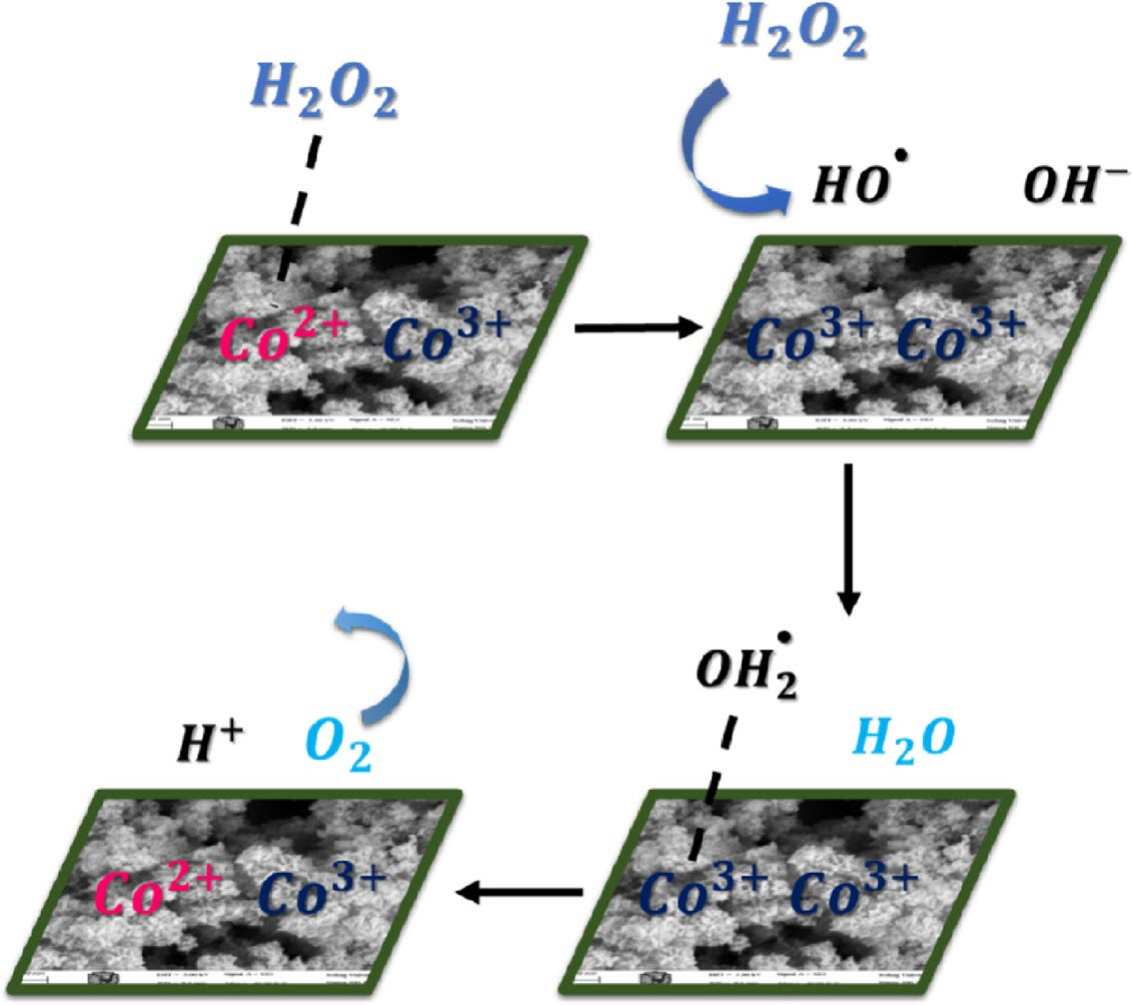
Supposed radical mechanism of the decomposition of H_2_O_2_ by green-synthesized Co_3_O_4_ catalysts.

In the radical mechanism (Fig. 10), H_2_O_2_ interacts with a metal ion (M^n+^) in the oxide lattice, generating a hydroxyl radical (HO^•^) as depicted in reaction 14. Subsequently, HO^•^ reacts with H_2_O_2_ to produce peroxide radical ($\:{\mathrm{O}\mathrm{H}}_{2}^{{\bullet\:}}$) (reaction 15). In the last step, $\:{\mathrm{O}\mathrm{H}}_{2}^{{\bullet\:}}$ reduces the oxidized metal ion ($\:{\mathrm{M}}_{\left(\mathrm{s}\mathrm{u}\mathrm{r}\mathrm{f}\right)\:}^{(\mathrm{n}+1)+}$) to give O_2_ (reaction 16).

14 $${\mathrm{M}}_{\left(\mathrm{s}\mathrm{u}\mathrm{r}\mathrm{f}\right)\:}^{\mathrm{n}+} + {\rm{ }}{{\rm{H}}_{\rm{2}}}{{\rm{O}}_{\rm{2}}} \to \:{\mathrm{M}}_{\left(\mathrm{s}\mathrm{u}\mathrm{r}\mathrm{f}\right)\:}^{(\mathrm{n}+1)+} + {\rm{ H}}{{\rm{O}}^ \bullet } + {\rm{ O}}{{\rm{H}}^ - }$$

15 $${{\rm{H}}_{\rm{2}}}{{\rm{O}}_{\rm{2}}} + {\rm{ H}}{{\rm{O}}^ \bullet } \to {\rm{ }}{{\rm{H}}_{\rm{2}}}{\rm{O }} + \:{\mathrm{O}\mathrm{H}}_{2}^{{\bullet\:}}$$

16 $$\:{\mathrm{M}}_{\left(\mathrm{s}\mathrm{u}\mathrm{r}\mathrm{f}\right)\:}^{\left(\mathrm{n}+1\right)+} \:+\:\:{\mathrm{O}\mathrm{H}}_{2}^{{\bullet\:}}\:\to\:\:{\mathrm{M}}_{\left(\mathrm{s}\mathrm{u}\mathrm{r}\mathrm{f}\right)\:}^{\mathrm{n}+}\:\to\: + {\rm{ }}{{\rm{H}}^ + } + {\rm{ }}{{\rm{O}}_{\rm{2}}}$$

**Fig. 11:**
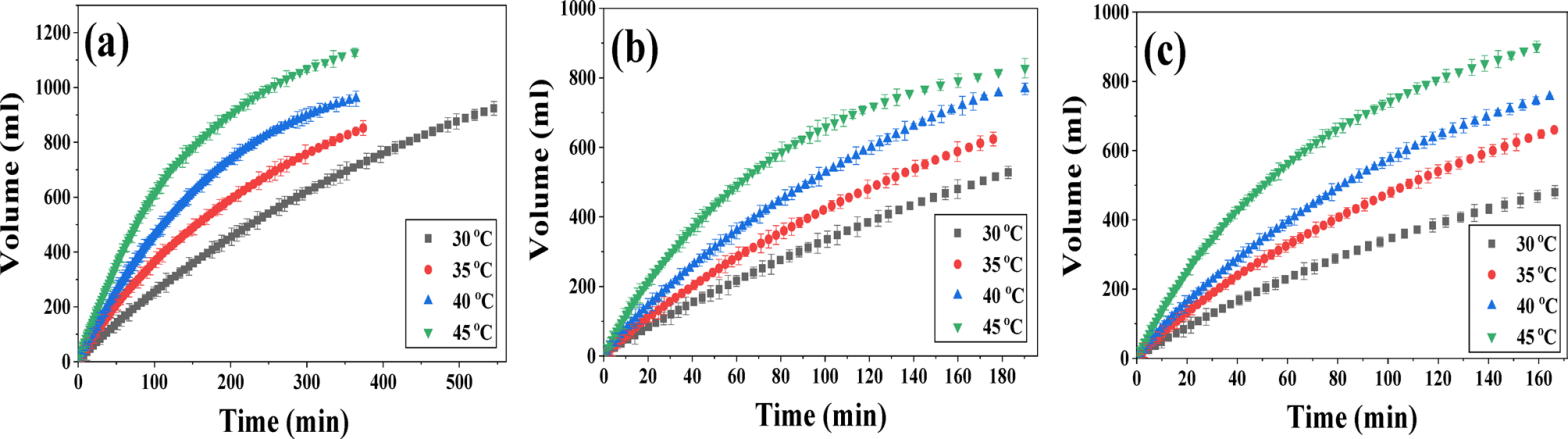
Abstracted data from V_O2_-time curves during H_2_O_2_ decomposition over Co_3_O_4_ catalysts (AHT **(a)**, AMW **(b)**, AWB **(c)**) at various reaction temperatures ranging from 30° to 45 °C under consistent parameters (10 mg of catalyst and 20 ml of 30% H_2_O_2_).

This process can occur on any catalyst capable of producing redox pairs $\:{\mathrm{M}}_{\left(\mathrm{s}\mathrm{u}\mathrm{r}\mathrm{f}\right)\:}^{\mathrm{n}+}/\:{\mathrm{M}}_{\left(\mathrm{s}\mathrm{u}\mathrm{r}\mathrm{f}\right)\:}^{\left(\mathrm{n}+1\right)+}$, such as in the spinel structure of Co_3_O_4_ NPs. In the case of Co_3_O_4_ catalysts, a key element in H_2_O_2_ decomposition is the presence of Co^2+^ ions on octahedral lattice sites, which can initiate a cyclic electron transfer process (redox reaction) at the catalyst surface^10,55^. This is caused by a larger number of Co^2+^ ions that are unfavorably located in tetrahedral sites within a weak electron donor system, compared to a smaller amount of Co^3+^ ions that are favorably situated in octahedral sites in a strong electron donor system. This supports the mechanism for H_2_O_2_ decomposition proposed by Schwab et al., which involves divalent transition metal ions within spinel lattices^55^. The decomposition reaction of H_2_O_2_ was examined at different reaction temperatures ranging from 30 to 45 °C (Fig. 11). As the temperature increased, the oxygen release rate rose, significantly accelerating the reaction. Noticeable differences in the degradation rates of H_2_O_2_ were observed among catalysts from different green routes, with degradation rates rising substantially as temperatures ranged from 30 to 45 °C. The integrated linear equation of the first-order equation and exponential form were presented in Eqs. 17 and 18.

17 $$\:\mathrm{ln}\left(\frac{\mathrm{a}}{\mathrm{a}-\mathrm{x}}\right)=\mathrm{k}\mathrm{t}$$

18 $$\:\frac{a}{a-x}={e}^{kt}$$

The non-linear equation (Eq. 4) of the first-order equation, which is abstracted from the exponential form of the linear equation (Eq. 18). The rate constant values of the catalysts were calculated by utilizing non-linear fitting of OriginPro software; these values are listed in Table 6. These values are utilized to calculate the activation energy (E_a_) from the plot between ln (k) and 1/T, as shown in Fig. S10 by applying the Arrhenius equation (Eq. 5). The activation energy values of the obtained catalysts were ordered as the highest E_a_ of 39.66, 56.58, and 59.49 kJ/mol to AWB, AMW, and AHT, respectively. The lower value of E_a_ is 39.66 kJ/mol in the AWB sample, which is evidence of higher catalytic activity of H_2_O_2_ decomposition. The commercial Co_3_O_4_ demonstrates limited catalytic efficiency in the decomposition reaction of H_2_O_2_, with an evolution of 9.1 ml of O_2_ over a period of 100 min, as documented in^35^. Significantly, the catalysts synthesized in this study exhibited higher activity in comparison to the commercial Co_3_O_4_ by evolving a higher O_2_ volume in a shorter time. The commercial variant has a lower specific surface area of 2.3 m^2^g^− 1^, which led to lower activity in H_2_O_2_ decomposition. Furthermore, the enhanced performance of the synthesized catalysts may be attributed to their larger specific surface area resulting from various morphologies. The superior performance observed in AWB is due to its higher ratios of oxygen vacancies (O_lattice_/O_defect_ (%)) and Co^2+^/Co^3+^ compared to AMW and AHT, as evidenced by the XPS spectra. Additionally, the larger surface area of AWB (116 m^2^g^− 1^) relative to AMW (96 m^2^g^− 1^) and AHT (57 m^2^g^− 1^), which matches the descending order of catalytic performance.

**Table 6:** The rate constant values (k) of the prepared catalyst were derived from the decomposition of H_2_O_2_ across various reaction temperature ranges (30–45 °C).

	Rate constant (k)*10^−3^ (min^−1^)			
Catalysts	30 °C	35 °C	40 °C	45 °C
AHT	2.1	4.4	5.2	6.8
AMW	4.4	5.95	7.4	13.5
AWB	6.6	7.7	8.7	14.6

## Conclusion

Various approaches were employed for the green synthesis of Co_3_O_4_ NPs from aloe vera leaves. XRD analysis and N_2_-adsorption verify the nano-crystalline nature of Co_3_O_4_ catalysts. FESEM images reveal that the nanostructures exhibit a uniform spherical morphology in both AMW and AWB, whereas AHT images display flower-like structures with a minor presence of sheet-like formation. FT-IR analysis and XPS data indicate the coexistence of Co^3+^ and Co^2+^ ions, which confirms the spinel structure of the Co_3_O_4_ crystal lattice. The obtained Co_3_O_4_ catalysts demonstrate a dual function in two reactions: NaBH_4_ hydrolysis and H_2_O_2_ decomposition. In case of NaBH_4_ hydrolysis, AHT exhibited the highest catalytic performance with HGR of 4267 ml.min^− 1^.g^− 1^ at 45 °C. The reason is attributed to its smaller pore size, higher content of Co^2+^ ions, and distinct morphology compared to the other catalysts. The optimal catalyst (AHT) undergoes different experimental parameters to evaluate the performance of the catalyst on HGR values; these effects have a greater influence on HGR values. An increase in catalyst dosage and NaBH_4_ concentration resulted in higher HGR values; however, the presence of a basic medium (NaOH) at various concentrations caused a sharp decline in HGR values with increasing concentrations. Furthermore, the activity of AHT has a lower decrease during the reusability of the catalyst, with lower conversion from 1990 to 1265 ml.min^− 1^.g^− 1^ at 35 °C. In the other reaction, AWB showed superior catalytic activity toward H_2_O_2_ decomposition. These findings are correlated with a larger surface area and lower Co^2+^ ions content.

## Supplementary Information

Below is the link to the electronic supplementary material.

Supplementary Material 1

## Data Availability

All data supporting the findings of this study are available within the paper and its Supplementary Information.
