# Supplementary material for: Different green synthesis methods of Co3O4 NPs using aloe vera leaves: enhance H2 and O2 production from NaBH4 hydrolysis and H2O2 decomposition
Source: Sci Rep. 2026 Jul 24;16:23216. doi: 10.1038/s41598-026-62856-x (PMC13400610; doi:10.1038/s41598-026-62856-x)
Supplement: Supplementary file 1 — Supplementary Material 1 [file 41598_2026_62856_MOESM1_ESM.docx]

**Different Green Synthesis Methods of Co_3_O_4_ NPs Using Aloe Vera Leaves: Enhance H_2_ and O_2_ Production from NaBH_4_ Hydrolysis and H_2_O_2_ Decomposition**

Simon W. Samouel^1^, Tarek T. Ali ^1^, Bahaa M. Abu-Zied ^2^, Hatem A. Mahmoud ^1,*^

^1^Chemistry Department, Sohag University, 82524 Sohag, Egypt

^2^Chemistry Department, Assiut University, 71516 Assiut, Egypt

Corresponding author: Hatem A. Mahmoud

Email address: hatem.elnakeeb@science.sohag.edu.eg


**Fig. S1:** N_2_ adsorption isotherms (a) and distribution of particle sizes (b) for green-synthesized catalysts.

**Fig. S2:** The survey of all synthesized catalysts, which only contain Co, O, C, and N.


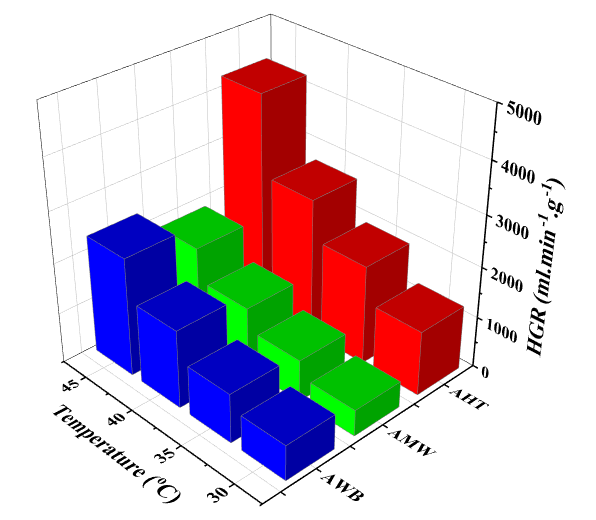
**Fig. S3:** HGR values of various methods for synthesizing Co_3_O_4_ NPs using aloe vera extract as an eco-friendly approach.

**Fig. S4:** plot of the natural logarithm of the rate constant (k) against the reciprocal of temperature (1/T) to determine the activation energy (E_a_) of the synthesized catalysts in the hydrolysis reaction of sodium borohydride (NaBH_4_): AHT (a), AMW (b), and AWB (c).

**Langmuir-Hinshelwood Kinetic Model Fit:** $\mathbf{C}_{\mathbf{t}}$ **vs Time (min)**

**Fig. S5:** A plot showing C_t_ versus time based on the zero-order kinetic model fit of the AHT catalyst to determine the reaction order by examining the effect of temperature (30 °C (a), 35 °C (b), 40 °C (c), and 45 °C (d)).

**Langmuir-Hinshelwood Kinetic Model Fit:** $\mathbf{C}_{\mathbf{t}}$ **vs Time (min)**

**Fig. S6:** Langmuir-Hinshelwood kinetic model fit of AHT catalyst for the investigation of catalytic performance with temperature effects of 30 °C (a), 35 °C (b), 40 °C (c), and 45 °C (d).

**Fig. S7:** Plot of ln(k) and ln(catalyst weight) for determination of the reaction order in catalyst weight effect (AHT) in catalytic hydrolysis of NaBH_4_.

**Fig. S8:** XRD analysis of AHT catalyst reveals the cause of the decline in catalytic activity during NaBH_4_ hydrolysis following the reusability process, from the fresh catalyst through to 5^th^ cycles.


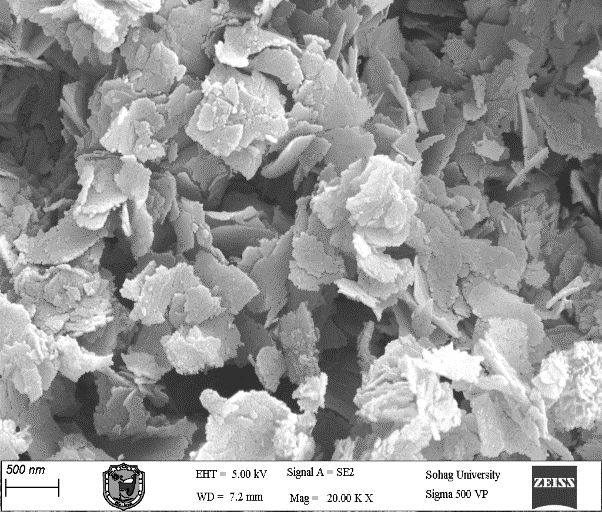

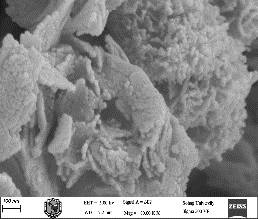

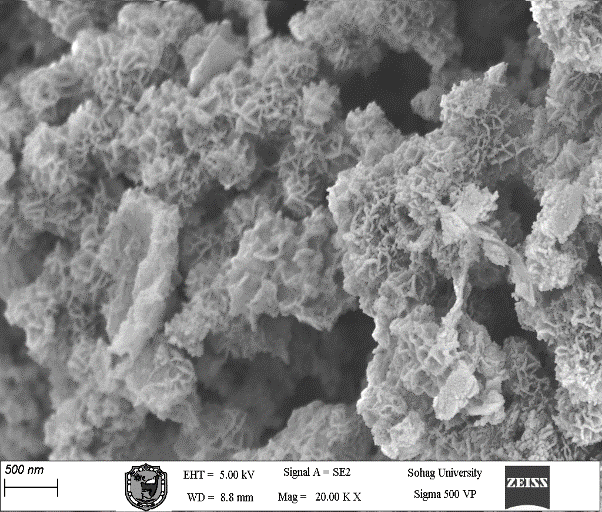

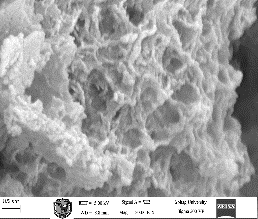


**After** $\mathbf{5}^{\mathbf{th}}$ **cycle**

$\boldsymbol{5}^{\boldsymbol{th}}$**cycle**

**Fresh catalyst**

**Fig. S9:** FE-SEM analysis was conducted to examine the morphological changes in AHT catalyst, aiming to elucidate the reasons for the decline in catalytic activity during NaBH_4_ hydrolysis after repeated reuse from the fresh catalyst to 5^th^ cycles.

**Fig. S10:** plot of the natural logarithm of the rate constant (k) against the reciprocal of temperature (1/T) to determine the activation energy (E_a_) of the synthesized catalysts in the decomposition reaction of hydrogen peroxide (H_2_O_2_): AHT (a), AMW (b), and AWB (c).

**Table S1:** Kinetic parameters of NaBH_4_ hydrolysis by using AHT catalyst for zero-order reaction model.

| Zero-order reaction | | |
| --- | --- | --- |
| T (°C) | **K (mol.l^-1^.min^-1^)** | **R^2^** |
| 30 | 0.01757 | 0.9933 |
| 35 | 0.02428 | 0.9947 |
| 40 | 0.0379 | 0.9934 |
| 45 | 0.03841 | 0.9912 |

**Table S2:** Kinetic parameters of NaBH_4_ hydrolysis by using AHT catalyst for the Langmuir-Hinshelwood kinetic model.

| Zero-order reaction | | | |
| --- | --- | --- | --- |
| T (°C) | **Ka** | **K (mol.l^-1^.min^-1^)** | **R^2^** |
| 30 | 65.90 | 0.00739 | 0.9999 |
| 35 | 100.21 | 0.0108 | 0.9996 |
| 40 | 77.50 | 0.0152 | 0.9989 |
| 45 | 3419.80 | 0.02068 | 0.9868 |
